# A mechanical lumped-element model of the human middle ear for bone conduction hearing

**DOI:** 10.21203/rs.3.rs-6262568/v1

**Published:** 2025-04-25

**Authors:** Xiying Guan

**Affiliations:** Department of Communication Sciences and Disorders, Department of Bioengineering, Wayne State University, Detroit, Michigan 48202

## Abstract

Bone conduction (BC) is an important modality of hearing. It enables us to differentiate conductive and sensorineural hearing loss, perceive sounds despite a disabled middle ear, and listen to conversation and music privately without blocking the ear canal. Yet the mechanism underlying BC is not fully understood mainly because the bone-conducted vibrations in the skull simultaneously stimulate the outer ear, the middle ear, and the cochlea. The nature of the parallel stimulation on those interconnected parts makes it difficult to contemplate the dynamics in each compartment and the influences they impose on each other.

In the present study, a computational lumped-element human ear model for BC is developed. The model comprises lumped mechanical components – masses, springs and dampers – to represent structures such as eardrum, ossicles, ligaments, joints, and cochlear fluid. The parameters of those components are determined by fitting the simulated ossicular vibrations to the measured counterparts reported by Stenfelt et al., the most extensive BC middle-ear dataset.

The results show that the model-predicted vibrations of the umbo and stapes generally match the experimental results not just in the normal ear condition but also after various perturbations such as adding mass on the eardrum and separating the incudostapedial joint.

It is believed this is the first lumped-element model that can correctly simulate the vibrations of the human middle ear in BC. The model can serve as the bedrock not only for better understanding the dynamics of the entire ear in BC but also for developing new diagnostics for middle-ear conditions and assisting design of novel hearing prostheses.

## 1. Introduction

Most of the everyday sounds we hear are vibrations transmitted by air particles from the sound source to our eardrums, relayed by the ossicles in the middle ear, and received by the fluid in the cochlea where the sound sensory cells are located. The modality of hearing the airborne sounds is known as air conduction (AC) hearing. Yet when there are vibrations in our body or skull, they can be transmitted to the ears by the bones surrounding the ears and evoke hearing, and this modality of hearing is known as bone conduction (BC) hearing. Because the bone near the ear is rigid, when it vibrates, the air in the ear canal, the ossicles in the middle ear, and the fluid in the inner ear are set into vibration at the same time. While this parallel stimulation makes BC hearing more resilient to ear diseases than AC hearing, it poses a greater challenge to understand the transmission of sound in the ear during BC stimulation. The number of analytical modeling work on the human ear for BC^1-8^ is substantially less than that for AC^9-21^. And the previous BC models were not fully validated against objective, dynamic measurements including the ossicle’s vibrations and intracochlear pressures in BC that were published more recently^2,22-27^.

BC is of great importance. The BC hearing test is used daily in clinics to differentiate conductive and sensorineural hearing loss and thus help pinpoint the cause of the hearing loss^28-31^. BC is the only natural way of hearing when the ear canal or middle ear is severely impaired^31-33^. BC hearing devices are favored over AC devices in sports and military operations when private communications are needed but the ear canal needs to stay open^34^. BC devices are a better option for communication than AC devices when the user’s head needs to be insulated from the surroundings and there is hence little room for AC devices (e.g. diver performing underwater tasks and healthcare workers wearing personal protective equipment^35^). Despite BC’s existing and potential applications, its underlying mechanisms remain elusive.

The prevailing theory is that BC hearing is contributed by three distinct mechanisms, and their superposition determines what we hear in BC. The first is the compression-expansion of the air in the external ear canal resulting from the out-of-phase vibration of the cartilaginous section of the canal, making the air particles vibrate and leading to hearing in the manner similar to AC^36-38^. Yet the most recent experiment on this topic shows that the ear-canal’s contribution to BC hearing in unoccluded human ears is only 5-15 dB^37^. The second mechanism is the compression-expansion of the bone enclosing the inner-ear fluid, producing a net movement in the fluid which stimulates the sensory cells^5,38,39^. But recent measurements indicate that in BC the bone encapsulating the fluid moves as a rigid body over an extended range of frequencies, leaving this mechanism palpable only at very high frequencies^23^. Those experimental work suggest that BC hearing perhaps is contributed mainly by the third mechanism – the inertias of the ossicles and the inner-ear fluid. The ossicles are loosely connected to the skull by soft tissues, and the fluid is in contact with two flexible windows. When the skull vibrates, the ossicles and the fluid, due to their inertias, can move relative to the skull and therefore propel the sensory cells in the cochlea. Despite its potency in BC, the inertia mechanism has not been fully understood due to the lack of theoretical works.

**Figure 1.**
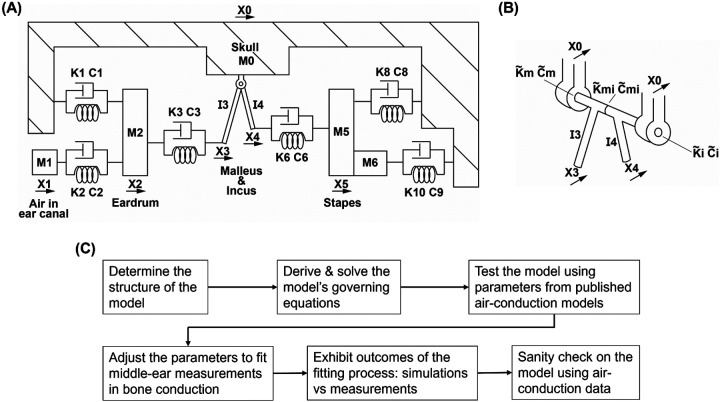
The schematic of the lumped-element model of the human ear (A), the detailed schematic of the malleus and incus (B), and the workflow from solving the model, constraining its parameters, to validating the simulations (C).

The present study devises a lumped-element model of the human ear to elucidate the ossicular component of the BC inertial mechanism (the structure and the development of the model are displayed in Fig. 1). The mechanical properties used in the model are initially adopted from previous AC human ear models and then adjusted so the model-predicted vibrations of the ossicles in BC are comparable with experimental measurements. The measurements used in the comparison were those reported by Stenfelt et al. in 2002^25^, the richest BC middle-ear dataset including the vibrations of the umbo and the stapes observed in intact ears and after various perturbations. The model in the present study is perhaps the first that can correctly predict the vibrations of the human ossicles in BC not just in the normal condition but also under different perturbed ones. We may for the first time have an analytical tool to comprehend an important mechanism underlying BC hearing.

## 2. Methods

### 2.1 Structure of the model

Figure 1A shows the lumped element model of the human ear in the present study. The mass M0 represents the skull surrounding the ear. $M_{1}$ represents the mass of air in the ear canal, which is coupled to the mass of the eardrum $M_{2}$ by the spring $K_{2}$ and the dashpot $C_{2}$. $C_{1}$ and $K_{1}$ represent the damping and the elasticity of the eardrum, suspending the mass of the eardrum from the skull.

The malleus and incus are represented by two rigid-body pendulums, which are hinged to the skull and can only rotate relative to the skull and relative to each other along the conventional anterior-posterior axis. Their moments of inertia about the rotation axis are denoted as $I_{3}$ and $I_{4}$, respectively.

As shown in Fig.1B, when the malleus and incus rotate, they need to overcome the rotational stiffness and rotational damping imposed by their suspension ligaments, which are represented by ${\tilde{K}}_{m}$, ${\tilde{C}}_{m}$, ${\tilde{K}}_{i}$, and ${\tilde{C}}_{i}$. The malleus is coupled with the incus by rotational spring ${\tilde{K}}_{mi}$ and rotational damping ${\tilde{C}}_{mi}$.

$K_{3}$ and $C_{3}$ represent the portion of the eardrum connected with the tip of the malleus (umbo), and $K_{6}$ and $C_{6}$ represent the incudostapedial joint.

$M_{5}$ embodies the stapes, and $K_{8}$ and $C_{8}$ represent its annular ligament. $M_{6}$ represents the mass of the fluid in the cochlea, sitting between the stapes and the elastic round window membrane $K_{10}$. Experiments have shown that when the fluid moves relative to the wall of the cochlea, it is subjected to a resistive force^40^. This cochlear resistance is represented by $C_{9}$.

### 2.2 Kinematics of the model

$M_{0}$, $M_{1}$, $M_{2}$, and $M_{5}$ move horizontally with displacement $X_{0}$, $X_{1}$, $X_{2}$, $X_{5}$, respectively. The cochlear fluid $M_{6}$ undergoes the same displacement as $M_{5}$. $I_{3}$ (malleus) and $I_{4}$ (incus) swing like a pendulum: the hinged, superior ends move together with the skull with the horizontal displacement $X_{0}$; the unhinged, inferior ends sway, thus moving horizontally and vertically. Because the malleus and the incus are mostly vertically oriented and the displacements of their inferior ends are much smaller than the dimensions of those two ossicles, the vertical displacements are negligible, and motion of the inferior ends are approximated as their horizontal displacements noted as $X_{3}$ and $X_{4}$. Thus the angles of rotation of the malleus and incus, respectively, are

$$\theta_{3}=\left(X_{3}-X_{0}\right)∕L_{3}\;\text{and}\;\theta_{4}=\left(X_{4}-X_{0}\right)∕L_{4},$$

where $L_{3}$ and $L_{4}$ are the distances from the rotation axis to the tips of the long process of those two bones.

### 2.3 Kinetics and governing equations of the model

For each mass and moment-of-inertia, a kinetic equation can be written to describe the relationship between an external sinusoidal driving force acting upon it, the restoring and damping forces, and the resulting vibration displacement (X) or angel (θ). In the following, the formation of such equation for M2 and I3 are given in details to exemplify the process.

Use ∑F=ma, the kinetic equation of $M_{2}$ is

$$F_{2}+C_{3}({\dot{X}}_{3}-{\dot{X}}_{2})+K_{3}(X_{3}-X_{2})+C_{2}({\dot{X}}_{1}-{\dot{X}}_{2})+K_{2}(X_{1}-X_{2})+C_{1}({\dot{X}}_{0}-{\dot{X}}_{2})+K_{1}(X_{0}-X_{2})=M_{2}{\ddot{X}}_{2},$$

where $F_{2}$ is an external, sinusoidal driving force on $M_{2}$. The equation can be rearranged as

(eq1) $$M_{2}{\ddot{X}}_{2}-C_{1}{\dot{X}}_{0}-C_{2}{\dot{X}}_{1}+(C_{1}+C_{2}+C_{3}){\dot{X}}_{2}-C_{3}{\dot{X}}_{3}-K_{1}X_{0}-K_{2}X_{1}+(K_{1}+K_{2}+K_{3})X_{2}-K_{3}X_{3}=F_{2}.$$

Similarly, the kinetic equations of $M_{1}$ and $M_{5}+M_{6}$ are

(eq2) $$M_{1}{\ddot{X}}_{1}+C_{2}{\dot{X}}_{1}-C_{2}{\dot{X}}_{2}+K_{2}X_{1}-K_{2}X_{2}=F_{1},\;\text{and}$$

and

(eq3) $$(M_{5}+M_{6}){\ddot{X}}_{5}-(C_{8}+C_{9}){\dot{X}}_{0}-C_{6}{\dot{X}}_{4}+(C_{6}+C_{8}+C_{9}){\dot{X}}_{5}-(K_{8}+K_{9})X_{0}-K_{6}X_{4}+(K_{6}+K_{8}+K_{9})X_{5}=F_{5}.$$

Each of those equations consists of an inertial component (product of mass and acceleration) and a few restoring and damping forces (imposed by springs and dashpots) balanced by an external force. The kinetic equation of the skull $M_{0}$ follows the same pattern. But since its inertial component ($M_{0}{\ddot{X}}_{0}$) is much bigger than its associated restoring and damping forces, the latter can be neglected and the equation for $M_{0}$ can thus be simplified as

(eq4) $$M_{0}{\ddot{X}}_{0}=F_{0},$$

where $F_{0}$ is an external sinusoidal driving force on the skull, providing the stimulation for BC.

To derive the kinetic equation for $I_{3}$, we use ∑τ=Iα and can write down

$$F_{3}L_{3}+{\tilde{C}}_{mi}({\dot{\theta }}_{4}-{\dot{\theta }}_{3})-{\tilde{C}}_{m}{\dot{\theta }}_{3}+C_{3}({\dot{X}}_{2}-{\dot{X}}_{3})L_{3}+K_{mi}(\theta_{4}-\theta_{3})-K_{m}\theta_{3}+K_{3}(X_{2}-X_{3})L_{3}=I_{3}{\ddot{\theta }}_{3},$$

where $F_{3}$ is an external sinusoidal driving force on the lower tip of $I_{3}$. Plug in $\theta_{3}$ and $\theta_{4}$ and rearrange the equation, the kinetic equation of $I_{3}$ becomes

(eq5) $$-\frac{I_{3}}{{L_{3}}^{2}}{\ddot{X}}_{0}+\frac{I_{3}}{{L_{3}}^{2}}{\ddot{X}}_{3}+\left(\frac{{\tilde{C}}_{mi}}{L_{3}L_{4}}-\frac{{\tilde{C}}_{m}+{\tilde{C}}_{mi}}{{L_{3}}^{2}}\right){\dot{X}}_{0}-C_{3}{\dot{X}}_{2}+\left(C_{3}+\frac{{\tilde{C}}_{m}+{\tilde{C}}_{mi}}{{L_{3}}^{2}}\right){\dot{X}}_{3}-\frac{{\tilde{C}}_{mi}}{L_{3}L_{4}}{\dot{X}}_{4}+\left(\frac{{\tilde{K}}_{mi}}{L_{3}L_{4}}-\frac{{\tilde{K}}_{m}+{\tilde{K}}_{mi}}{{L_{3}}^{2}}\right)X_{0}-\;\;K_{3}X_{2}+\left(K_{3}+\frac{{\tilde{K}}_{m}+{\tilde{K}}_{mi}}{{L_{3}}^{2}}\right)X_{3}-\frac{{\tilde{K}}_{mi}}{L_{3}L_{4}}X_{4}=F_{3}.$$

Similarly, the kinetic equation of $I_{4}$ is

(eq6) $$-\frac{I_{4}}{{L_{4}}^{2}}{\ddot{X}}_{0}+\frac{I_{4}}{{L_{4}}^{2}}{\ddot{X}}_{4}+\left(\frac{{\tilde{C}}_{mi}}{L_{3}L_{4}}-\frac{{\tilde{C}}_{i}+{\tilde{C}}_{mi}}{{L_{4}}^{2}}\right){\dot{X}}_{0}-\frac{{\tilde{C}}_{mi}}{L_{3}L_{4}}{\dot{X}}_{3}+\left(C_{6}+\frac{{\tilde{C}}_{i}+{\tilde{C}}_{mi}}{{L_{4}}^{2}}\right){\dot{X}}_{4}-C_{6}{\dot{X}}_{5}+\left(\frac{{\tilde{K}}_{mi}}{L_{3}L_{4}}-\frac{{\tilde{K}}_{i}+{\tilde{K}}_{mi}}{{L_{4}}^{2}}\right)X_{0}-\;\;\frac{{\tilde{K}}_{mi}}{L_{3}L_{4}}X_{3}+\left(K_{6}+\frac{{\tilde{K}}_{i}+{\tilde{K}}_{mi}}{{L_{4}}^{2}}\right)X_{4}-K_{6}X_{5}=F_{4},$$

where $F_{4}$ is an external driving force on the lower tip of $I_{4}$.

Lastly, to solve $X_{0}∼X_{5}$ for any given external force(s) $F_{0}∼F_{5}$, we put equations 1-6 in the matrix form

(eq7) $$\{-\omega^{2}[M]+i\omega [C]+[K]\}[X]=[F],$$

where


$$\;[M]=\left[\begin{array}{cccccc} M_{0} & \; & \; & \; & \; & \;\\ \; & M_{1} & \; & \; & \; & \;\\ \; & \; & M_{2} & \; & \; & \;\\ -\frac{I_{3}}{{L_{3}}^{2}} & \; & \; & \frac{I_{3}}{{L_{3}}^{2}} & \; & \;\\ -\frac{I_{4}}{{L_{4}}^{2}} & \; & \; & \; & \frac{I_{4}}{{L_{4}}^{2}} & \;\\ \; & \; & \; & \; & \; & M_{5}+M_{6} \end{array}\right],\;\;[C]=\left[\begin{array}{cccccc} \; & C_{2} & -C_{2} & \; & \; & \;\\ -C_{1} & -C_{2} & C_{1}+C_{2}+C_{3} & -C_{3} & \; & \;\\ \frac{{\tilde{C}}_{mi}}{L_{3}L_{4}}-\frac{{\tilde{C}}_{m}+{\tilde{C}}_{mi}}{{L_{3}}^{2}} & \; & -C_{3} & C_{3}+\frac{{\tilde{C}}_{m}+{\tilde{C}}_{mi}}{{L_{3}}^{2}} & -\frac{{\tilde{C}}_{mi}}{L_{3}L_{4}} & \;\\ \frac{{\tilde{C}}_{mi}}{L_{3}L_{4}}-\frac{{\tilde{C}}_{i}+{\tilde{C}}_{mi}}{{L_{4}}^{2}} & \; & \; & -\frac{{\tilde{C}}_{mi}}{L_{3}L_{4}} & C_{6}+\frac{{\tilde{C}}_{i}+{\tilde{C}}_{mi}}{{L_{4}}^{2}} & -C_{6}\\ -(C_{8}+C_{9}) & \; & \; & \; & -C_{6} & C_{6}+C_{8}+C_{9} \end{array}\right],\;[K]=\left[\begin{array}{cccccc} \; & K_{2} & -K_{2} & \; & \; & \;\\ -K_{1} & -K_{2} & K_{1}+K_{2}+K_{3} & -K_{3} & \; & \;\\ \frac{{\tilde{K}}_{mi}}{L_{3}L_{4}}-\frac{{\tilde{K}}_{m}+{\tilde{K}}_{mi}}{{L_{3}}^{2}} & \; & -K_{3} & K_{3}+\frac{{\tilde{K}}_{m}+{\tilde{K}}_{mi}}{{L_{3}}^{2}} & -\frac{{\tilde{K}}_{mi}}{L_{3}L_{4}} & \;\\ \frac{{\tilde{K}}_{mi}}{L_{3}L_{4}}-\frac{{\tilde{K}}_{i}+{\tilde{K}}_{mi}}{{L_{4}}^{2}} & \; & \; & -\frac{{\tilde{K}}_{mi}}{L_{3}L_{4}} & K_{6}+\frac{{\tilde{K}}_{i}+{\tilde{K}}_{mi}}{{L_{4}}^{2}} & -K_{6}\\ -(K_{8}+K_{9}) & \; & \; & \; & -K_{6} & K_{6}+K_{8}+K_{9} \end{array}\right],\;\;[X]=\left[\begin{array}{c} X_{0}\\ X_{1}\\ X_{2}\\ X_{3}\\ X_{4}\\ X_{5} \end{array}\right],\;\text{and}\;[F]=\left[\begin{array}{c} F_{0}\\ F_{1}\\ F_{2}\\ F_{3}\\ F_{4}\\ F_{5} \end{array}\right].$$

This allows us to solve [X] for a given [F]. The middle-ear vibrations in BC can therefore be simulated by setting $[F]=\left[\begin{array}{c} F_{0}\\ 0\\ 0\\ 0\\ 0\\ 0 \end{array}\right]$, where $F_{0}$ is the harmonic driving force on the skull. If needed, the AC responses can be simulated by setting $[F]=\left[\begin{array}{c} 0\\ F_{1}\\ 0\\ 0\\ 0\\ 0 \end{array}\right]$, where $F_{1}$ is the driving force on the air in the ear canal and the corresponding stimulating sound pressure is $F_{1}∕A$, where A is the ear canal’s cross sectional area.

### 2.3 Parameters in the model

**Figure 2.**
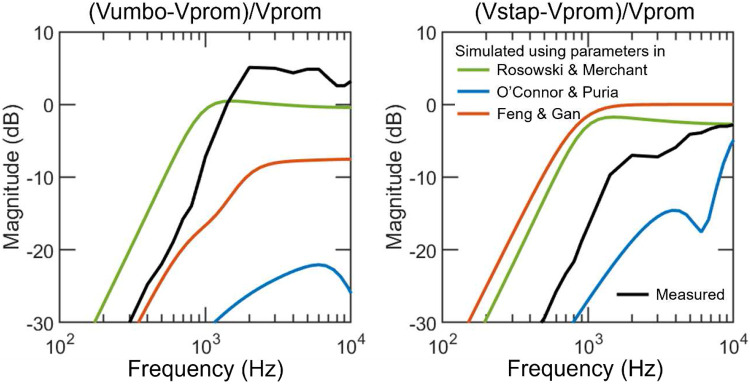
Comparison between the measured and the simulated umbo (left) and stapes (right) velocity magnitudes with respect to the promontory in human ears in bone conduction. The black lines represent the measurements in Stenfelt et al. (25); the blue lines represent model-simulated results using the parameters in O’Connor and Puria (16); the red lines represent simulated results using the parameters in Feng and Gan (12); the green lines represent simulated results using the parameters in Rosowski and Merchant (17).

The parameters in [M], [C], and [K] are determined through two steps. First, the parameters from three independent, validated lumped-element human ear models for AC^12,16,17^ are adopted and tested in the present model. These parameters are summarized in Table 1, and the simulated BC umbo and stapes vibrations based on each parameter set are displayed in Fig. 2 and described in section 3.1.

Second, since it produces better results than the other two, the set of parameters adopted from Rosowski and Merchant^17^ is adjusted manually until the model-predicted results match Stenfelt et al.’s BC middle-ear measurements (mean results) under various ear conditions reasonably well. The ear conditions and the measurements used to constrain the parameters are summarized in Table 2. The head-to-head comparisons between the measurements and the simulations made by the adjusted model are shown and discussed in section 3.2.

## 3. Results

### 3.1 BC umbo- and stapes-vibrations simulated using published, unadjusted parameters

Figure 2 shows the model-predicted velocities of the umbo (Vumbo ${\dot{X}}_{3}$) and the stapes (Vstap ${\dot{X}}_{5}$) relative to the skull/promontory velocity (Vprom ${\dot{X}}_{0}$) in BC using the parameters adopted from the three published AC models (Table 1). The velocity of the umbo and the stapes simulated using parameters from Rosowski and Merchant^17^ and Feng and Gan^12^ display a pattern similar to that measured by Stenfelt et al.: they increase with frequency below 1000 Hz and plateau at higher frequencies. Although there is quite a gap between the prediction and the measurement, the tryout suggests that the model’s framework is likely correct and a closer fit after adjusting the parameters is plausible.

### 3.2 BC umbo- and stapes-vibrations simulated using fitted parameters

Since it produces the most palpable results, the set of parameters based on Rosowski and Merchant^17^ is chosen for further adjustment, with the goal of matching the simulated results with the BC umbo- and stapes-vibration data in Stenfelt et al.^25^ – a total of 12 mean curves measured under the normal and manipulated conditions (Table 2). The adjusted parameters derived from the fitting process are listed in the last column of Table 1.

**Figure 3.**
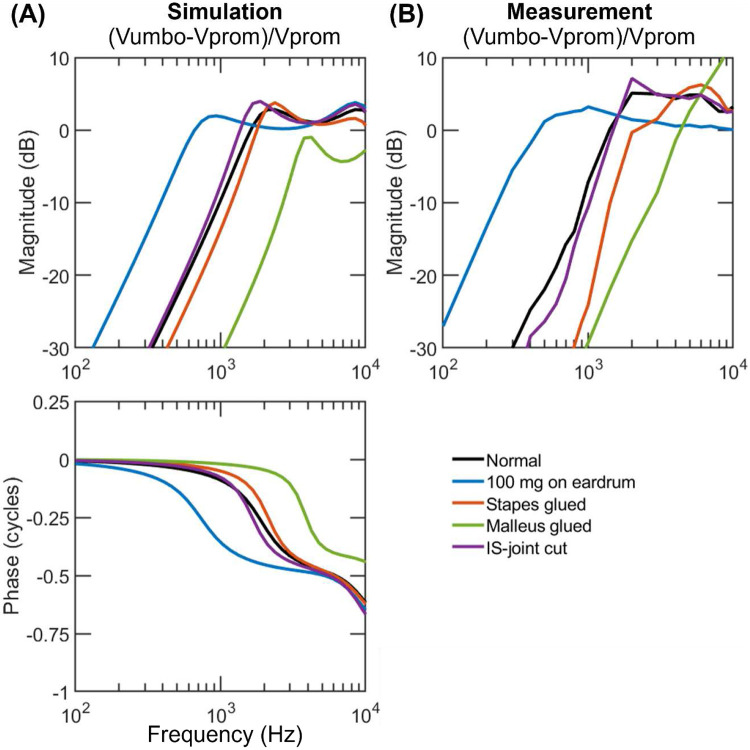
Simulated (A) and measured (B) umbo velocities with respect to the promontory in human ears under normal (black lines) and perturbed ear conditions (color lines). The simulations are performed using the fitted parameters. The measured magnitudes are from Stenfelt et al. (25), and the corresponding phases were not reported. IS joint: incudostapeidal joint.

**Figure 4.**
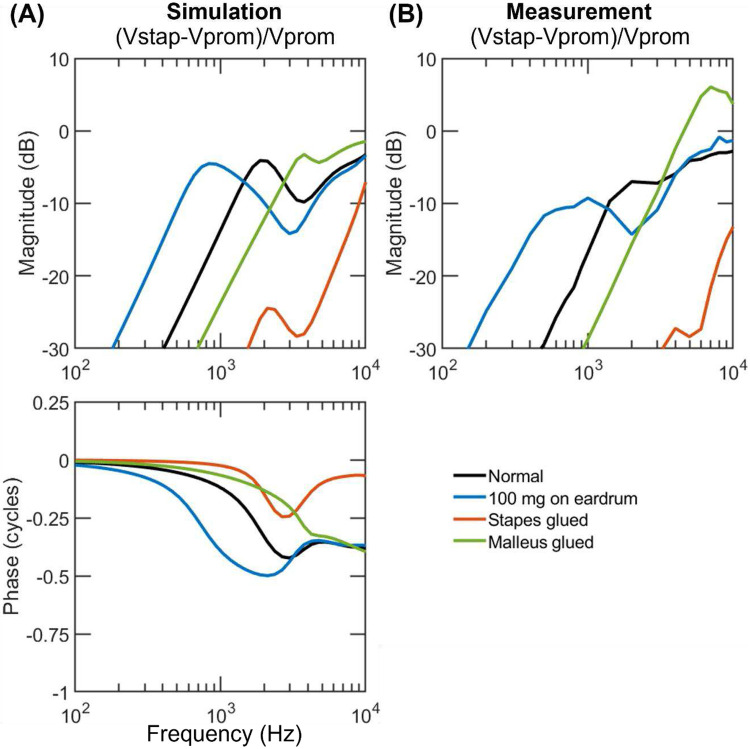
Simulated (A) and measured (B) stapes velocities with respect to the promontory in human ears under normal (black lines) and perturbed ear conditions (color lines). The simulations are performed using the fitted parameters. The measured magnitudes are from Stenfelt et al. (25), and the corresponding phases were not reported.

**Figure 5.**
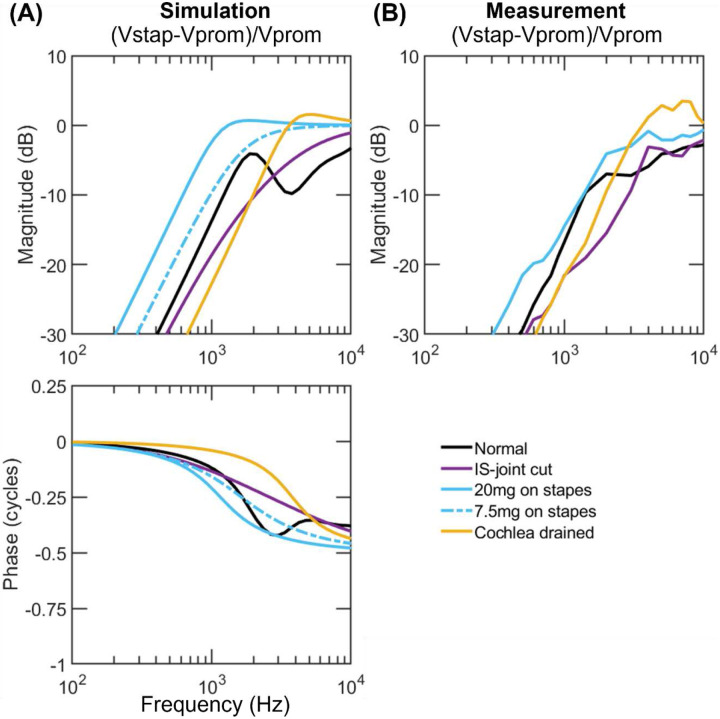
Simulated (A) and measured (B) stapes velocities with respect to the promontory in human ears under the normal condition (black lines), after the separation of incudostapedial joint (purple lines), and under the subsequent perturbations (cyan and yellow lines). The measured magnitudes are from Stenfelt et al. (25), and the corresponding phases were not reported. IS joint: incudostapeidal joint.

Figures 3-5 exhibit the velocities of the umbo and the stapes relative to the promontory ($\frac{Vumbo-Vprom}{Vprom}$ and $\frac{Vstap-Vprom}{Vprom}$) simulated with the adjusted parameters in comparison to their experimental counterparts; the simulated phases are also provided while the phase data from the experiments are not since they were not reported in Stenfelt et al^25^. Supplementary figures 1-3 display the ratio $\frac{Vumbo}{Vprom}$ and $\frac{Vstap}{Vprom}$. In each plot, the velocity under the normal ear condition is represented by the black line, while those under manipulated conditions are represented by colored lines. The last row in Table 2 describes how the manipulations are simulated in the model.

Figure 3A shows that when the skull vibrates at very low frequencies (< 300 Hz), the movement of the umbo relative to the skull in a normal ear is extremely small (black line), as if the ossicles are rigidly coupled to the skull. As the frequency increases, the relative motion increases rapidly and peaks near 2 kHz, the first resonant frequency observed in the result; from there it decreases slightly and then rises again toward a second resonance near 10 kHz. Those two resonances are what keep the curve slightly above 0 dB at 2-10 kHz. The mean measurement (black line in Fig. 3B) displays the same trend but with a flatter curve at those high frequencies. The less varying response is most likely due to averaging. Many of the individual measurements (Fig. 6b in Stenfelt et al.^25^) display a second resonance in the band of 3-10 kHz and the frequency of it varies from ear to ear so that the mean appears flat in this region.

Figure 3A also shows the simulated effects of four perturbations on the umbo movement. The model predicts that separating the incudostapedial joint hardly affects the umbo’s motion in BC (purple line); adding mass on the eardrum lowers the first resonant frequency and thus increases the umbo’s vibration substantially at low frequencies (blue line); gluing the malleus head greatly reduces the motion at low frequences and shifts the first resonance to a higher frequency (green line); gluing the stapes also reduces the umbo motion but to a modest degree. These simulated effects generally agree with the measurements shown in Fig. 3B. An exception is that after gluing the malleus in real ears the umbo’s motion exceeds the normal above 6 kHz (green line in Fig. 3B), suggesting the manubrium may undergo bending at those high frequencies once the head of the malleus is fixed. Such mechanism does not exist in the present model.

Figure 4A displays the simulated stapes velocity with respect to the skull in the normal and three perturbed conditions. The normal stapes motion increases quickly with frequency until the first resonance at 2 kHz, then decreases to a local minimum at 3-4 kHz, and rises at higher frequencies (black line). The overall trend is similar to that seen in umbo, but the stapes vibrates at a smaller magnitude than the umbo above 1 kHz.

The model predicts that adding mass on the eardrum lowers the stapes’ first resonant frequency (blue line in Fig. 4A), elevating its movement substantially below 1.2 kHz and lowering it at 1.2-4 kHz; gluing the malleus attenuates the stapes motion below 2.5 kHz but causes an increase at higher frequencies (green line); gluing the stapes greatly suppresses its movement at all the tested frequencies (red line). These simulated results in general match the experimental ones shown in Fig. 4B except that gluing the malleus in real ears causes a greater increase in stapes motion at high frequencies.

Figure 5A shows the simulated stapes velocity relative to the skull after the separation of the incudostapedial joint and three subsequent, independent perturbations (the normal result is also provided for comparison). Dislocating the joint removes the 2 kHz-resonance seen in the normal response, decreasing the stapes movement at 1-2 kHz and increasing it modestly at higher frequencies (purple line vs black line). Adding 20 mg to the stapes thereafter engenders a shallow resonant peak near 1.5 kHz and leads to a greater stapes motion than normal across the frequencies (cyan solid line); adding 7.5 mg to the stapes produces a similar but smaller effect (cyan chain line). Lastly, draining the cochlea results in a resonance near 4 kHz, reducing the stapes movement below 2 kHz and increasing it at higher frequencies (yellow line) in comparison to normal. These predicted effects are generally consistent with the measurements shown in Fig. 5B except that the measurement with the 20-mg weight in real ears is closer to the simulation with 7.5 mg than 20 mg.

In sum, the results in Figs.3-5 demonstrate that the model equipped with the fitted parameters predicts the umbo and stapes vibrations in BC fairly well under not just a few but all the tested conditions (five for umbo and seven for stapes). This outcome suggests the model can be an analytical solution for the middle-ear dynamics in BC. However, to gain more confidence in the model, its parameters derived from the fitting process need to be examined carefully.

### 3.3. Sanity check

Two procedures are used to check if the fitted parameters are reasonable: first, they are directly compared with those in the previous models; second, they are used in the present model to simulate middle-ear vibrations in AC, and the results are compared with published AC measurements.

**Figure 6.**
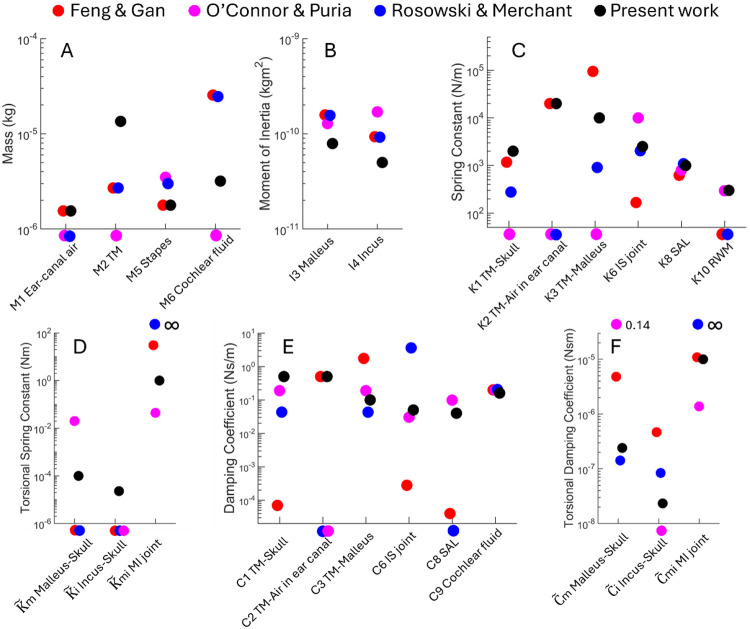
Comparison between the fitted parameters in the present study and those in the previous air-conduction models including Feng and Gan (12), O’Connor and Puria (16), and Rosowski and Merchant (17). TM – tympanic membrane; MI joint – incudomalleolar joint; IS joint – incudostapedial joint; SAL – stapedial annular ligament; RWM – round window membrane.

Figure 6 displays the fitted values of all the components in the present model (black dots) and those adopted from the previous work (colored dots; exact numbers are listed in Table 2). The parameters that are zero or not existing (equivalent to zero) are plotted below the horizontal axis. By glancing at the parameters from the three previous models, it is immediately noticed that the values of a component can group closely (e.g. M5, I3, I4, K8, C9) but can also differ greatly (e.g. K3, K6, C1, C6, C8, Cm). Most of the parameters in the present model are within or adjacent to the span of variation in the previous parameters.

One parameter standing out is the mass of the eardrum (M2 in Fig. 6A), which is five times larger than the typical value. M2 greatly influences the amplitude and frequency of the first resonance of the middle ear, and moreover, the measurements shown in Figs.3-5 seem to demand a heavier eardrum as using a normative M2 and tweaking other parameters to compensate could not fit the data across the board.

**Figure 7.**
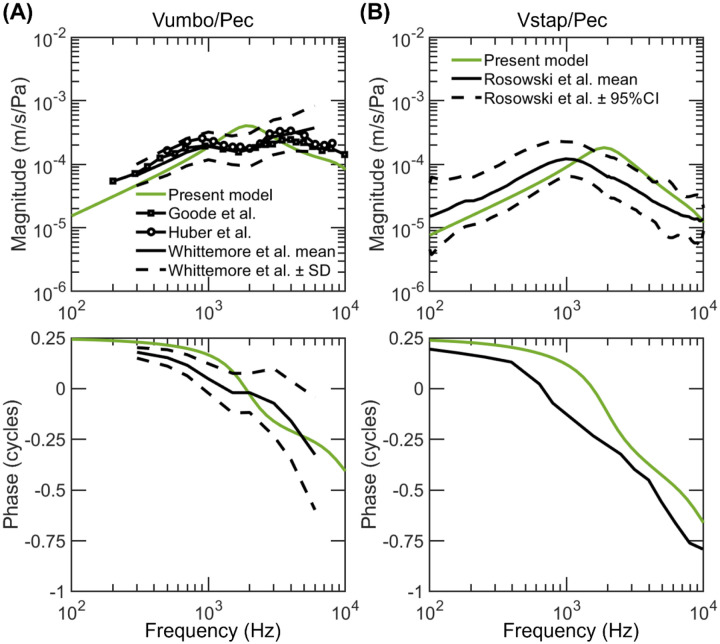
Comparison between the simulated and the measured umbo (A) and stapes (B) velocity with respect to the ear-canal pressure in human ears in air conduction. The green lines represent the model-predicted results using the fitted parameters. In (A) the black line with squares represents the mean of 64 ears in Goode et al. (41), the black line with circles the mean of 90 ears in Huber et al. (42), the solid black line the mean of 80 ears with ± one standard deviation (dashed black lines) in Whittemore et al (43). In (B) the solid black line represents the grand mean of 13 study means with ± 95% confidence intervals (dashed black lines) in Rosowski et al (44).

Figure 7 compares simulated and measured AC umbo^41-43^ and stapes^44^ velocity with respect to ear canal sound pressure (Pec) under the normal condition. The simulated results (green line in Fig.7A and &7B) are in general within the normative boundaries of the measurements except that the first resonant frequency in both simulations is near 2 kHz while that in the measurements is near 1 kHz.

The umbo and stapes in the AC simulation resonate around 2 kHz because the 2-kHz resonance is an intrinsic property of the model, determined and inherited from that in the BC measurements (see black lines in Figs. 3B and 4B). Perhaps the mechanical properties of the specimens in Stenfelt et al.’s BC experiments^25^ differ slightly from those in the AC experiments^41-44^, leading to a higher resonant frequency. The variance in the middle ear’s first resonance is not uncommon. Voss et al. showed in their AC measurement that this resonance locates between 1-2 kHz with an average near 1.5 kHz^45^.

In short, none of the fitted parameters is outlandish, and the simulated AC umbo and stapes vibrations are reasonable. Those results, together with the BC comparisons shown above, suggest that the structure and the mechanical properties of the present model mimic those of a real ear with some fidelity. The following discusses the model’s potential applications, limitations, and prospective improvements.

## 4. Discussion

### 4.1 The model’s potential applications

The above results suggest that the present model can predict the eardrum and ossicular movements in BC. There are at least four areas in which we can further leverage the model. First, we can use it to better understand the middle-ear mechanism of BC hearing by manipulating individual components in the model and studying the effects on the stapes-skull differential motion (representing the net movement of the cochlear fluid in BC). We can, for instance, change the moment of inertia of the malleus (I3) and see how that affects the stapes movement.

Second, we can use the model to predict how middle-ear pathologies change BC hearing. We have shown how perturbations such as ossicular fixation or additional mass affect BC, and we can also simulate the effects of other lesions such as a loose/disrupted incudomalleolar joint, a rigidified incudostapedial joint, or a stiffened eardrum (as a result of scarring), etc. It has been thought that the middle ear has a limited role in BC hearing, but there were early studies recognizing that anomalies such as extra mass on the eardrum or ossicles increase low-frequency sensitivity in BC, a possible etiology for hyperacusis^38,46-49^. We can use the present model to help identify conditions that change BC sensitivity and better understand the underlying mechanism.

Third, we can utilize the model to help develop new objective diagnostics for middle ear disorders. Some ossicular pathologies, such as malleus fixation^50,51^ and incudostapedial joint stiffening^52^, alter the sound-induced vibration of the umbo or eardrum substantially. But such measurement on patients is technically challenging in part because a probe microphone, sometimes together with a sound-delivery apparatus, need to be inserted in the ear canal, squeezing the space needed for the vibration measurement (when a laser Doppler vibrometer is used for example^43,51^). By contrast, measuring BC-induced umbo or eardrum vibration is much more feasible as the stimulus can be delivered anywhere on the skull and the ear canal is free of obstruction. Since the present model can help predict the ailments to which the BC umbo and eardrum vibrations are sensitive, it will thus be an essential tool when developing the diagnostic.

Fourth, we can use this model to help develop novel ear devices. For instance, fully implantable hearing aids will count on implantable miniature microphones. Some of those microphones are by design anchored to or placed on ossicles, leveraging the ossicular vibration as the input^53-56^. The present model can predict how such microphone – simulated as an additional spring and/or mass in the model – affects ossicular motion and thus help determine the physical constraints in the design to ensure the desired input level can be met. Another crucial component for implantable hearing aids is a minute shaker, which directly stimulates an ossicle. The model can conveniently simulate such stimulation at different places in the ossicular chain (e.g. turn on F4 in eq. 7 if the stimulation is at the tip of incus) and predict the efficacy of the implant. Moreover, the model can help assess how the implanted hardware affects one’s residual hearing for AC and BC.

Taken together, the present model is valuable not only because it can improve our knowledge about BC hearing but also for its potential use in creating new diagnostics and hearing devices.

### 4.2 Limitations and future work

There are a few limitations in the current model. First, the fitting process was done by adjusting the model parameters manually. Although this approach allows a better understanding of how each parameter influences the simulation, the end result may not be the optimal solution. The simulated results could be closer to the experimental data if an automated fitting program was used.

Second, the malleus and the incus are attached to the skull by ligaments, therefore, they may move not only rotationally but translationally with respect to the skull. The latter is not incorporated in the present model because we assume the translational stiffness imposed by the ligaments is much higher than their torsional stiffness. But the translational motion may exist at high frequencies (where stiffness in general plays a minor role) and give rise to another resonance. In Stenfelt et al.^25^ many of the ears display a second resonance varying between 3 and 10 kHz, a feature absent from the current simulations (the model predicts a higher resonance near 10 kHz). This discrepancy can be better perceived by comparing the simulated normal curve of $\frac{Vumbo}{Vprom}$ in supplemental Fig. 1A with the individual measurements in Fig. 6a in Stenfelt et al^25^. The second resonance might arise from the malleus and incus’ translational movements.

Third, the simple representation of the cochlea in the model seems adequate for simulating middle-ear vibrations but may not fully capture the dynamics within the cochlea in BC. For example, the intracochlear sound pressures, the drive of the cochlear traveling waves, can be produced by not just the footplate motion relative to the bony capsule of the cochlea but also the rigid-body movement of the capsule itself^2,5^. The latter cannot be simulated in the model because the corresponding mechanism is not included.

Lastly, the current model only incorporates the inertia mechanism of BC and does not include the BC compressional phenomenon speculated by some researchers. It has been postulated that the cartilage in the ear canal and the bony shell of the cochlea during BC may undergo compression-expansion, generating pressure waves^5,36,38^. But recent experiments show that when the ear canal is open the contribution of the ear-canal compression to BC hearing is trivial in human and animal ears^37,57^ and that the compression of the cochlea is insignificant below 10 kHz in human ears^23^.

The priorities of the model’s next iteration therefore should focus on adding the cochlear boundary, allowing the translational movement of the malleus and the incus (relative to the skull), and implementing a parameter-optimizing algorithm.

## 5. Conclusion

Here we presented a lumped-element model of the human ear that can predict the middle-ear vibrations in BC. The simulated results are comparable to the experimental data across various ear conditions. The model has a vast potential in applications like helping understand the middle-ear BC mechanism and abnormal BC hearing, aiding develop new diagnostics for middle-ear pathologies, and assisting the design of new hearing prostheses.

## Figures and Tables

**Table 1.** Adopted parameters from the previous air-conduction models and the final fitted parameters in the present model. The method of how each parameter from the previous models is adopted is indicated in the brackets where the letters are the ones used in the original work except the following constants: the length of the malleus $L_{m}=6.28\times 10^{\text{-}3}\;m$, the length of the incus $L_{i}=4.83\times 10^{\text{-}3}\;m$, the area of the footplate $A_{fp}=3.2\times 10^{\text{-}6}\;m^{2}$, and the area of the tympanic membrane $A_{tm}=6\times 10^{\text{-}5}\;m^{2}$.

	Symbol and unit in present model	Feng and Gan 2004 (12)	O’Connor and Puria 2008 (16)	Rosowski and Merchant 1995 (17)	Final fitted value in present model
Mass of air in ear canal:	M1(kg)	$1.55\times 10^{\text{-}6}\;(M_{1})$	n/a*	n/a*	1.55×10^−6^
Coupling of ear-canal air and TM	K2(N∕m) C2(Ns∕m)	$20,001\;(K_{2})$ $0.5\;(C_{2})$	n/a	n/a	20,001 0.5
Mass of TM (kg)	M2(kg)	$2.7\times 10^{\text{-}6}\;(M_{2})$	n/a	$2.7\times 10^{\text{-}6}\;(L_{T1}A_{tm}^{2})$	1.35×10^−5^
Coupling of TM and skull	K1(N∕m) C1(Ns∕m)	$1,175\;(K_{1})$ $0.00007\;(C_{1})$	n/a $0.189\;(Z_{0tm}A_{tm}^{2}∕2)$	$276.92\;((1∕C_{T2})A_{tm}^{2})$ $0.0432\;(R_{T2}A_{tm}^{2})$	2,000 0.1
Coupling of TM and malleus	K3(N∕m) C3(NS∕M)	$94,740\;(K_{3})$ $1.74\;(C_{3})$	n/a $0.189\;(Z_{0tm}A_{tm}^{2}∕2)$	$909.09\;(1∕C_{TS}^{M})$ $0.043\;(R_{TS}^{M})$	10,000 0.1
Moment of inertia of malleus	$I3\;({\mathrm{kgm}}^{2})$	$1.58\times 10^{\text{-}10}\;(M_{3}L_{m}^{2})$	$1.28\times 10^{\text{-}10}\;(M_{m}L_{m}^{2})$	$1.56\times 10^{\text{-}10}\;((L_{MI}^{M}∕2)L_{m}^{2})$	7.9×10^−11^
Coupling of malleus and skull	${\tilde{K}}_{m}\;(\mathrm{Nm},\mathrm{torsional})$ ${\tilde{C}}_{mi}\;(\mathrm{Nsm},\mathrm{torsional})$	n/a $4.81\times 10^{\text{-}6}\;(C_{4}L_{m}^{2})$	$0.02\;(K_{m}L_{m}^{2})$ $0.14\;(R_{m}L_{m}^{2})$	$0\;(L_{m}^{2}∕(C_{MI}^{M}∕2))$ $1.42\times 10^{\text{-}7}\;((R_{MI}^{M}∕2)L_{m}^{2})$	1×10^−4^ 2.405×10^−7^
Malleus-incus joint	${\tilde{K}}_{mi}\;(\mathrm{Nm},\mathrm{torsional})$ ${\tilde{C}}_{mi}\;(\mathrm{Nsm},\mathrm{torsional})$	$30.33\;(K_{5}L_{m}L_{i})$ $1.09\times 10^{\text{-}5}(C_{5}L_{m}L_{i})$	$0.0443\;(K_{imj}L_{m}L_{i})$ $1.38\times 10^{\text{-}6}(R_{imj}L_{m}L_{i})$	infinite (fused) infinite (fused)	1 1×10^−5^
Moment of inertia of incus	$I4\;({\mathrm{kgm}}^{2})$	$9.33\times 10^{\text{-}11}\;(M_{4}L_{i}^{2})$	$1.70\times 10^{\text{-}10}\;(M_{i}L_{i}^{2})$	$9.21\times 10^{\text{-}11}\;((L_{MI}^{M}∕2)L_{i}^{2})$	5×10^−11^
Coupling of incus and skull	${\tilde{K}}_{i}\;(\mathrm{Nm},\mathrm{torsional})$ ${\tilde{C}}_{i}\;(\mathrm{Nsm},\mathrm{torsional})$	n/a $4.67\times 10^{\text{-}7}\;(C_{7}L_{i}^{2})$	n∕a	$0\;(L_{i}^{2}∕(C_{MI}^{M}∕2))$ $8.40\times 10^{\text{-}8}\;((R_{MI}^{M}∕2)L_{i}^{2})$	2.3×10^−5^ 2.335×10^−8^
Incus-stapes joint	K6(N∕m) C6(Ns∕m)	$167\;(K_{6})$ $0.00028\;(C_{6})$	$1\times 10^{4}\;(K_{isj})$ $3.04\times 10^{\text{-}2}\;(R_{isj})$	$2,041\;(1∕C_{J})$ $3.6\;(R_{J})$	2500 0.05
Mass of stapes	M5(kg)	$1.78\times 10^{\text{-}6}\;(M_{5})$	$3.50\times 10^{\text{-}6}\;(M_{s}A_{fp}^{2})$	$3\times 10^{\text{-}6}\;(L_{S})$	1.78×10^−6^
Stapedial annular ligament	K8(N∕m) C8(Ns∕m)	$623\;(K_{8})$ $0.00004\;(C_{8})$	$798.63\;(K_{al}A_{fp}^{2})$ $0.0986\;(R_{al}A_{fp}^{2})$	$1,089\;((1∕C_{AL})A_{fp}^{2})$ $0\;(R_{AL}A_{fp}^{2})$	1000 0.04
Mass of cochlear fluid	M6(kg)	$2.55\times 10^{\text{-}5}\;(M_{6})$	n∕a	$2.46\times 10^{\text{-}5}\;(L_{c}A_{fp}^{2})$	3.1875×10^−6^
Damping of cochlear fluid	C9(Ns∕m)	$0.2\;(C_{9}+C_{10})$	$0.1962\;(R_{c}A_{fp}^{2})$	$0.2048\;(R_{c}A_{fp}^{2})$	0.16
Stiffness of round window	K10(N∕m)	n/a	$295.80\;(K_{rw}A_{fp}^{2})$	n/a	300
Mass of the skull	M1(kg)	n/a	n/a	n/a	5

* Substituted by the value of M1 in Feng and Gan when simulating the results in Fig. 2.

**Table 2.** Measurements and ear conditions in Stenfelt et al. (25) for constraining the parameters and validating the simulations in the model. The last row shows how each condition is simulated in the model. IS joint: incudostapeidal joint.

	Before IS-joint cut				After IS-joint cut		
	Normal	Stapes glued	Malleus glued	Mass loading 100 mg on umbo	IS-joint cut	Mass loading 20 mg on stapes	Cochlea drained
$\frac{V_{umbo}-V_{promontory}}{V_{promontory}}$(mean)	●	●	●	●	●		
$\frac{V_{footplate}-V_{promontory}}{V_{promontory}}$(mean)	●	●	●	●	●	●	●
Simulated with	The final parameters in Table 1	Increase K8 to 5×10^4^	Increase ${\tilde{K}}_{m}$ to 0.8	Add 100 mg to M2 and increase C1 to 0.4	Set K8=0 and C6=0	Add 20 mg to M5	Set C9=0 and K10=0

## Data Availability

The datasets generated in the current study are available in the figshare repository and can be accessed by https://figshare.com/s/075ade687d51cd4213ee and are available from the corresponding author on reasonable request.
